# Supplementary material for: Mobile sex-tech apps: How use differs across global areas of high and low gender equality
Source: PLoS One. 2020 Sep 11;15(9):e0238501. doi: 10.1371/journal.pone.0238501 (PMC7486082; doi:10.1371/journal.pone.0238501)
Supplement: S1 Survey Tool — (DOCX) [file pone.0238501.s005.docx]

**S1 Survey Tool: Full Survey Instrument**

The Internet has become a major source of information and sharing among people of all ages, genders and orientations worldwide. Technology is now tightly interwoven with our daily lives, and we use it to connect with and manage many of the things that are most important to us.

**For some of us, this includes love and sex.** In this survey, we are interested in learning more about how people use technology to learn more about sex, to connect with potential partners, to keep their relationships exciting or to track various aspects of their private lives. By conducting research on the impact of technology on people’s sexual relationships, we can better understand how these technologies are being used, how they benefit their users and how they might be improved.

1. How old are you?
   1. Under 18
   2. 18-20
   3. 21-24
   4. 25-34
   5. 35-44
   6. 45-54
   7. 55+
2. In what country do you live?
3. Is this your country of birth?
   1. Yes
   2. No
4. Are you? (multiple choice)
   1. Woman
   2. Genderqueer/Nonbinary
   3. Man
   4. Not listed
   5. Prefer not to say
5. Are you transgender?
   1. Yes
   2. No
   3. Don’t know
   4. Prefer not to say
6. How would you describe your current sexual orientation?
   1. Straight/Heterosexual
   2. Lesbian/Gay/Homosexual
   3. Bisexual/Pansexual
   4. Queer
   5. Asexual
   6. None of the above
   7. Prefer not to say
7. Have you ever had consensual sexual intimacy with another person (e.g., sexual touching, oral sex, intercourse)?
   1. Yes
   2. No
   3. Prefer not to say
8. Which types of sexual relationships have you had in the past? Check all that apply.
   1. Within a monogamous relationship
   2. Within an open/polyamorous relationship (with more than one person)
   3. Outside of a relationship (e.g., one-night stands, hook-ups, friends with benefits)
   4. Other
   5. None of the above
   6. Prefer not to say
9. Which type of sexual relationships do you currently have? Check all that apply.
   1. A monogamous relationship
   2. An open/polyamorous relationship (with more than one person)
   3. Casual sex relationships (e.g., one-night stands, hook-ups, friends with benefits)
   4. Other
   5. I am not currently in a sexual relationship
   6. Prefer not to say
10. Have you ever used an app to learn more about sex or sexual intimacy?
    1. Yes
    2. No
11. Which app(s)? [open text box]
12. Why did you use these apps? What did you hope to learn or find out? (Insert open text box)
13. Have you ever used an app to find a sexual partner? Check all that apply.
    1. Yes, for one night stands/hookups (one date)
    2. Yes, for a short-term relationship (a few meetings/dates)
    3. Yes, for a friends-with-benefits relationship (no romantic connection, but regular/frequent sex)
    4. Yes, for chatting and sexting, but not to arrange a meeting/date/hook-up
    5. Yes, for a long-term relationship
    6. Other
    7. No, I have not used an app to find a sexual partner.
14. Have you ever used an app to track your sexual activity (such as recording the days you have sex, whether you had an orgasm, or any other aspect of the experience)?
    1. Yes
    2. No
15. Which app(s)? [open text box]
16. Have you ever used an app to track sexually transmitted infections (STIs)?
    1. Yes
    2. No
17. Which app(s)? [open text box]
18. Have you ever used an app to track sexual satisfaction (e.g., orgasms)?
    1. Yes
    2. No
19. Which app(s)? [open text box]
20. Have you ever sexted (sent someone content of a sexual nature) via text or an app?
    1. Yes
    2. No
21. If yes, check all that apply:
    1. Text
    2. Photos
    3. Audio/talking
    4. Video
    5. Other
22. If yes to either, which channel or app do you use for these? Check all that apply:
    1. The texting feature on my phone
    2. Facebook Messenger
    3. Google Hangout
    4. Twitter DMs
    5. Instagram DMs
    6. Whatsapp
    7. Snapchat
    8. Signal
    9. Other: write in
23. Have you used an app or mobile device to improve your sexual relationship with a partner?
    1. Yes
    2. No
24. Which app(s)? [open text box]
25. If yes, *how* did the app improve your sexual relationship with your partner? Check all that apply.
    1. The app helped you explore new sexual experiences, like toys or positions.
    2. The app helped you stay connected when you could not see each other in person.
    3. The app helped you learn about ways to have safer sex.
    4. The app helped you feel more comfortable with your body or sexuality.
    5. The app helped you feel more comfortable with your partner’s body.
    6. The app helped you learn what your partner finds arousing.
    7. The app helped your partner learn what you find arousing.
    8. The app helped you introduce other people into your sexual relationships.
    9. The app helped you learn about polyamory/open relationships/consensual non-monogamy.
    10. The app helped you feel more emotionally connected to your partner.
    11. The app did not help or wasn’t useful.
    12. The app did not help and it was detrimental to your sexual relationship.
    13. Other [open text box]
